# Massive Pleural Synovial Sarcoma Presenting With Obstructive Shock

**DOI:** 10.1002/ccr3.73498

**Published:** 2026-11-11

**Authors:** Shenda Hou, Meher Binte Ali, Mashal Ali, Ahmed Abdelwahed, Danish Jilani

**Affiliations:** ^1^ University of Maryland Medical Center Baltimore Maryland USA; ^2^ MountainView Regional Medical Center Las Cruces New Mexico USA; ^3^ University of Maryland Baltimore Maryland USA

**Keywords:** malignant neoplasm, pleura, sarcoma, shock

## Abstract

Primary pleural synovial sarcoma (PPSS) is an exceptionally rare and aggressive malignancy. We report a massive PPSS characterized by positive TLE1 immunostaining but negative SS18–SSX Fluorescence in situ hybridization (FISH). This case highlights the importance of integrating morphology, immunohistochemistry, and clinical context when molecular testing is inconclusive.

## 1 Introduction

Synovial sarcoma is a highly aggressive malignant neoplasm derived from undifferentiated mesenchymal cells [1]. It most commonly arises in the extremities, particularly near large joints in young adults. Intrathoracic synovial sarcoma has been associated with the highest case‐fatality rate (71.5%; 148/207 cases) and the lowest 5‐year overall survival (OS) rate (26.0%; 95% CI: 19.6%–32.9%) compared with other primary sites [2]. Reported median survival ranges from 14.5 to 50 months, with 5‐year survival rates varying from 0% in some series to approximately 30% in others [2].

Advances in molecular diagnostics have significantly improved the accuracy of synovial sarcoma diagnosis. Immunohistochemical staining for TLE1 and molecular confirmation of the t(X;18)(p11;q11) translocation resulting in the SS18–SSX fusion gene are considered highly sensitive and specific diagnostic tools [3, 4, 5].

## 2 Case History/Examination

A 40‐year‐old male with a history of childhood asthma, tobacco use (7 cigarettes per day for 15 years), heroin use, and occupational exposure to industrial waste—including possible heavy metals such as cadmium—while working as a trash collector for 10 years, presented with 1 week of progressive right flank pain and mild dyspnea at rest and with exertion. Imaging revealed multiple large, multiloculated, peripherally enhancing cystic masses occupying the majority of the right hemithorax with marked leftward mediastinal shift (Figures 1 and 2). Additional findings included a right upper lobe segmental pulmonary embolism, diffuse narrowing of the right pulmonary arterial tree, and severe compression of the pulmonary venous vasculature with a slitlike inferior vena cava (IVC). Severe apical‐predominant bullous emphysema was present bilaterally. Multifocal consolidation in the left lung raised concern for bronchopneumonia, and imaging findings were also suggestive of hepatic congestion.

**FIGURE 1:**
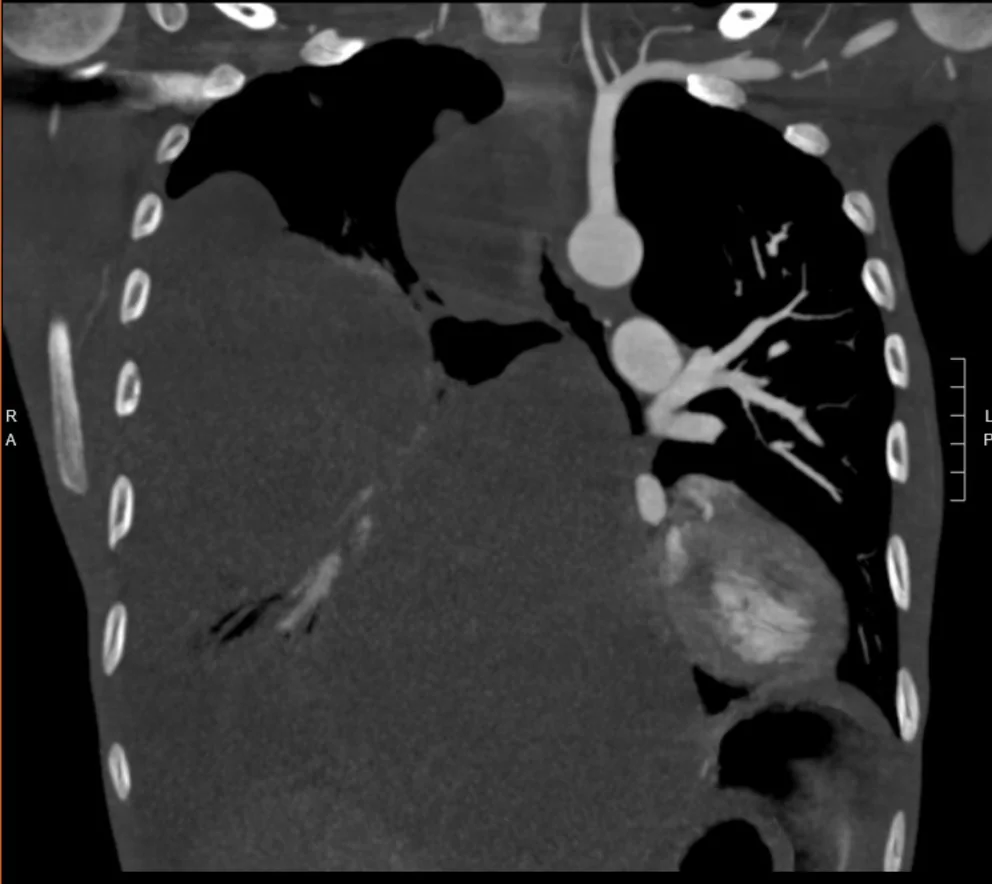
CT scan showing multiloculated pleural masses occupying the right hemithorax with left‐sided mediastinal shift.

**FIGURE 2:**
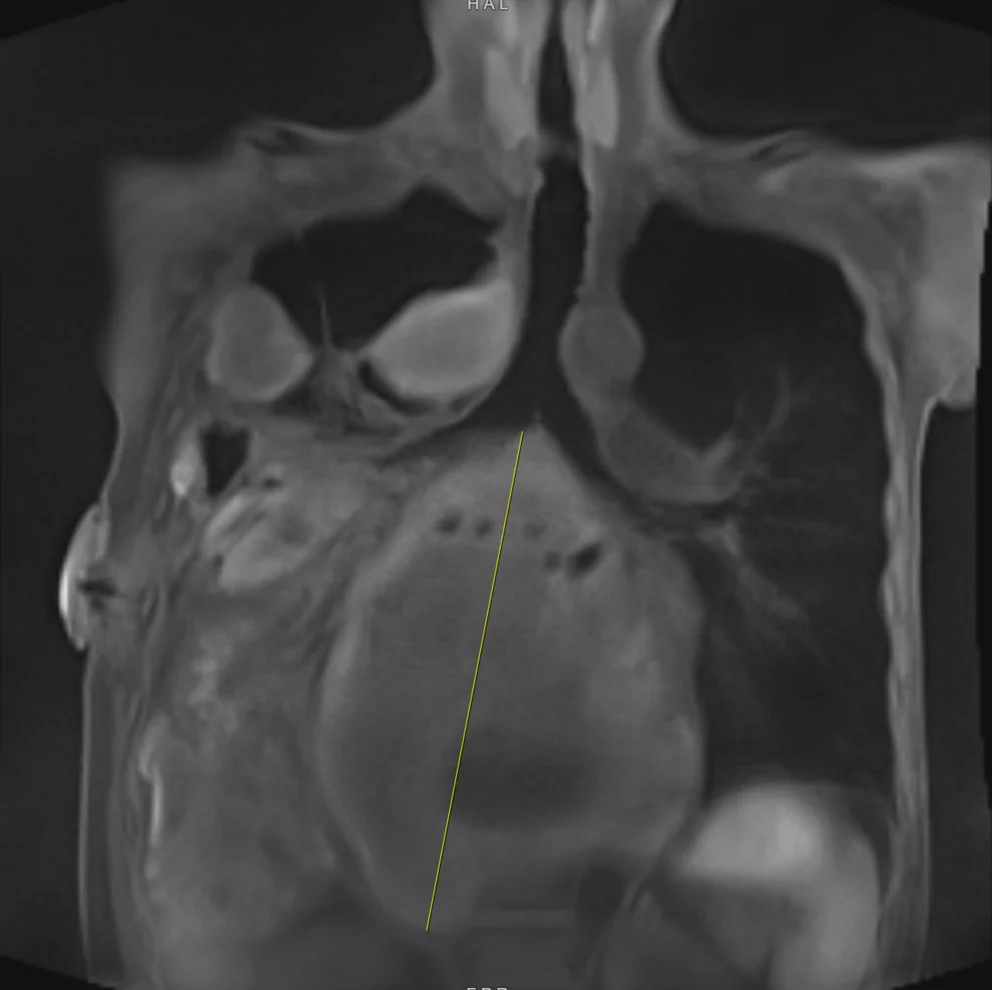
MRI showing multiple heterogeneous cystic/necrotic masses present throughout right hemithorax with a large mass present at the subcarinal region measuring approximately 14 × 8 × 19 cm.

Laboratory studies demonstrated elevated lactate, alpha‐fetoprotein (AFP), and carcinoembryonic antigen (CEA), raising concern for an underlying neoplastic process. The patient rapidly developed obstructive shock and respiratory failure requiring endotracheal intubation and vasopressor support.

## 3 Investigation/Treatment

The patient underwent thoracic debulking surgery, and tissue samples were obtained for histopathologic evaluation. Due to the markedly elevated AFP level, the patient's young age, and male sex, an aggressive germ cell tumor was initially suspected, and one cycle of etoposide and cisplatin was administered while awaiting biopsy results.

Histopathologic examination demonstrated a largely necrotic malignant neoplasm composed of a monomorphic spindle‐to‐epithelioid cell population arranged in interlacing fascicles, with hemangiopericytoma‐like vasculature (Figure 3A). Tumor cells were medium to large in size with vesicular chromatin and prominent nucleoli (Figure 3B). Frequent mitotic figures were identified. Immunohistochemical analysis showed that the neoplastic cells were positive for TLE1, CD99, TFE3, and BCL‐2 and negative for STAT6, CD20, PAX5, CD3, MUM1, pancytokeratin, CAM5.2, EMA, WT1, S100, and SOX10 (Figure 3C–E). These findings supported a diagnosis of synovial sarcoma involving the pleura.

**FIGURE 3:**
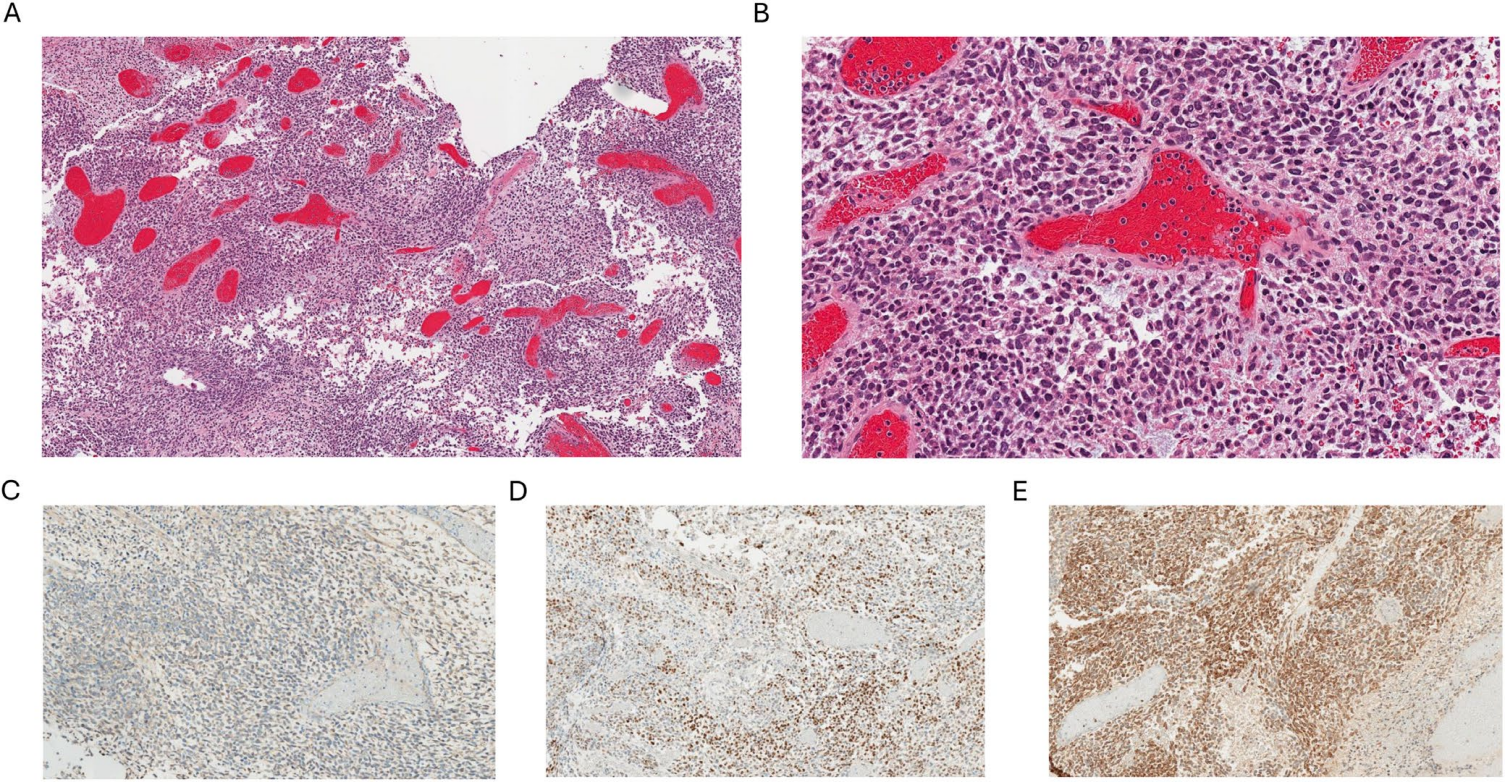
Pathology tumor imaging. (A) Low power HE staining showing a monomorphic spindle‐to‐epithelioid cell population arranged in interlacing fascicles, with hemangiopericytoma‐like vasculature. (B) High power HE staining showing tumor cells with vesicular chromatin and prominent nucleoli. (C) CD99 immunostaining. (D) TLE3 immunostaining. (E) BCL2 immunostaining.

Fluorescence in situ hybridization (FISH) analysis for SS18 rearrangement was performed at a reference laboratory and returned negative. Further imaging with MRI and FDG PET/CT demonstrated extensive right‐sided pleural‐based cystic and necrotic masses with intense peripheral hypermetabolic activity (SUVmax 10.5) and central photopenia, without evidence of distant metastatic disease (Figure 4).

**FIGURE 4:**
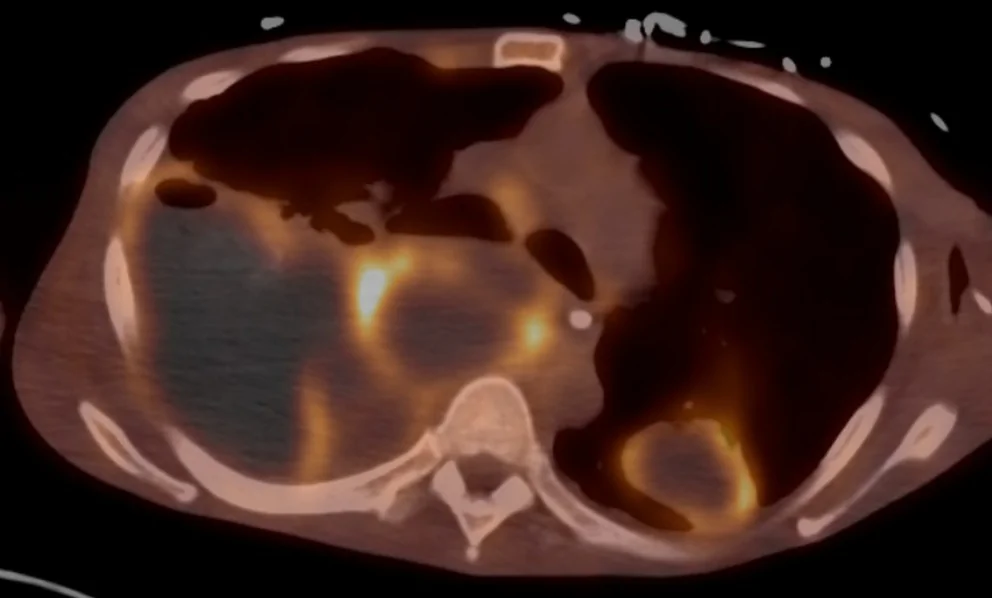
PET scan showing extensive right hemithorax cystic fluid‐filled/necrotic masses with intense peripheral metabolic activity.

Based on the clinical presentation, histomorphology, immunophenotype, and radiologic findings, a final diagnosis of primary pleural synovial sarcoma (PPSS) was established. The patient was initiated on AIM chemotherapy (doxorubicin, ifosfamide, and mesna), which was complicated by encephalopathy and neutropenia. Thoracic surgery was consulted; however, after a multidisciplinary discussion, surgical intervention was deemed excessively high‐risk with minimal anticipated therapeutic benefit given the extensive disease burden, ventilator dependence, and limited expected survival benefit.

The patient subsequently underwent three sessions of palliative thoracic radiation; however, further radiation was considered unlikely to provide benefit due to the predominantly cystic and necrotic composition of the tumor.

## 4 Outcome

A second cycle of chemotherapy at a reduced dose was planned, but the patient developed progressive respiratory failure and, after goals‐of‐care discussions with him, he opted to transition to comfort care and died approximately 5 weeks after presentation.

## 5 Discussion

Primary pleural synovial sarcoma (PPSS) is an exceptionally rare and aggressive malignancy, with approximately 100 cases reported in the literature [6]. Because of its rarity and nonspecific clinical presentation, diagnosis is often delayed until the disease has reached an advanced stage, which contributes to the poor overall prognosis. Patients typically present with symptoms related to mass effect, including chest pain, dyspnea, cough, or pleural effusion. Radiologically, these tumors frequently appear as large heterogeneous pleural‐based masses with areas of necrosis, hemorrhage, and cystic degeneration. Histologically, tumors have dense cellularity, interlacing fascicles, hyalinized stroma, and mast cell influx, with the majority demonstrating hemangiopericytoma‐like vasculature [7].

The etiology of PPSS remains poorly understood due to the rarity of the disease, and an association with environmental factors has not been clearly established. In our case, the patient demonstrated severe apical‐predominant bullous emphysema that appeared disproportionate to his relatively limited smoking history. This raises the possibility of additional contributing factors. Notably, the patient had chronic occupational exposure to waste materials. Cadmium, a heavy metal commonly present in industrial waste, has been associated with emphysematous lung injury [8]. Although this exposure may have contributed to the patient's pulmonary injury, current evidence regarding the role of environmental factors in the development of PPSS remains limited.

Optimal management strategies for PPSS remain undefined. A multimodal approach including surgery, chemotherapy, and radiotherapy has been proposed, although its efficacy remains uncertain [9]. Systemic therapy for advanced synovial sarcoma typically consists of doxorubicin alone or in combination with ifosfamide. Combination therapy may achieve higher response rates but is associated with increased toxicity, as observed in our case.

Several case reports have described long‐term survival following repeated surgical resections [10, 11]. In one reported case, a 39‐year‐old male with FISH‐negative disease underwent left extrapleural pneumonectomy and achieved 4‐year survival after multiple subsequent resections [11]. Although our patient might theoretically have benefited from aggressive surgical management, the technical complexity and high perioperative risk rendered surgery a less favorable option compared with systemic therapy.

According to the 5th edition of the WHO Classification of Soft Tissue and Bone Tumors, the essential diagnostic criteria for synovial sarcoma include compatible morphology and diffuse, strong nuclear immunostaining for TLE1 [12], both of which were present in this case. The SS18–SSX fusion oncogene drives tumorigenesis by linking SS18, a component of the mSWI/SNF (BAF) chromatin‐remodeling complex, to Polycomb repressive complex 1 (PRC1) target genes [13]. Detection of the SS18–SSX fusion is considered a specific molecular hallmark of synovial sarcoma, and FISH is commonly used in clinical practice with a reported sensitivity of 82%–97% [12]. However, the diagnostic accuracy of FISH compared with RT‐PCR remains debated [14, 15].

Rare cases of tumors with morphology and immunophenotype consistent with synovial sarcoma but negative SS18 FISH results have been reported [12, 16]. In several such cases, next‐generation sequencing identified novel or variant SS18–SSX translocations not detectable by conventional FISH assays [12, 16]. A similar mechanism may explain the findings in our patient. Continued investigation into variant translocations and refinement of molecular diagnostic assays are essential to improve the accuracy of synovial sarcoma diagnosis.

## Author Contributions

**Shenda Hou:** conceptualization, investigation, writing – original draft, writing – review and editing. **Meher Binte Ali:** conceptualization, investigation, writing – original draft, writing – review and editing. **Mashal Ali:** writing – original draft, writing – review and editing. **Ahmed Abdelwahed:** writing – original draft, writing – review and editing. **Danish Jilani:** writing – original draft, writing – review and editing.

## Funding

The authors have nothing to report.

## Consent

Written informed consent was obtained from the patient for publication of this case report and any accompanying images.

## Conflicts of Interest

The authors declare no conflicts of interest.

## Data Availability

Data sharing is not applicable to this article as no datasets were generated or analyzed during the current study.
